# Quantum Authentication Evolution: Novel Approaches for Securing Quantum Key Distribution

**DOI:** 10.3390/e26060447

**Published:** 2024-05-26

**Authors:** Hassan Termos

**Affiliations:** Lab-STICC, CNRS UMR 6285, ENSTA Bretagne, 2 Rue François Verny, CEDEX 09, 29806 Brest, France; hassan.termos@ensta-bretagne.fr

**Keywords:** quantum key distribution, BB84, SARG04, quantum bit error rate, mono-authentication

## Abstract

This study introduces a novel approach to bolstering quantum key distribution (QKD) security by implementing swift classical channel authentication within the SARG04 and BB84 protocols. We propose mono-authentication, a pioneering paradigm employing quantum-resistant signature algorithms—specifically, CRYSTALS-DILITHIUM and RAINBOW—to authenticate solely at the conclusion of communication. Our numerical analysis comprehensively examines the performance of these algorithms across various block sizes (128, 192, and 256 bits) in both block-based and continuous photon transmission scenarios. Through 100 iterations of simulations, we meticulously assess the impact of noise levels on authentication efficacy. Our results notably highlight CRYSTALS-DILITHIUM’s consistent outperformance of RAINBOW, with signature overheads of approximately 0.5% for the QKD-BB84 protocol and 0.4% for the QKD-SARG04 one, when the quantum bit error rate (QBER) is augmented up to 8%. Moreover, our study unveils a correlation between higher security levels and increased authentication times, with CRYSTALS-DILITHIUM maintaining superior efficiency across all key rates up to 10,000 kb/s. These findings underscore the substantial cost and complexity reduction achieved by mono-authentication, particularly in noisy environments, paving the way for more resilient and efficient quantum communication systems.

## 1. Introduction

Quantum computing has garnered substantial interest for its potential to outperform classical computers in tackling intricate computational tasks. Grover’s algorithm, for instance, demonstrates a quadratic speedup in searching unstructured databases compared to classical methods, offering significant advantages for optimization problems such as database search and cryptographic key cracking [1]. Similarly, Shor’s algorithm showcases exponential efficiency in factoring large integers, a task traditionally challenging for classical computers [2]. Its implications extend to cryptographic applications, particularly in solving the elliptic curve discrete logarithm problem, marking a notable advancement in security protocols [3]. Moreover, quantum devices show promise in simulating complex system dynamics, with implications across diverse fields from material science to drug discovery [4].

Recent breakthroughs have underscored quantum computing’s computational edge over classical counterparts, emphasizing its transformative potential across various domains. However, the practical realization of Shor’s algorithm poses significant concerns for internet security, as widely used cryptographic protocols rely on problems like integer factorization and discrete logarithm calculations. While current quantum computers lack the power to execute Shor’s algorithm efficiently, the trajectory of quantum computing advancement necessitates proactive measures to anticipate potential future cryptographic vulnerabilities [5,6].

The scientific community has responded to this challenge through multifaceted initiatives, including the development of quantum-resistant cryptographic algorithms within the realm of post-quantum cryptography (PQC). These algorithms are designed to address problems with exponential time complexity, offering resilience against both conventional and quantum computing paradigms [7,8]. Additionally, alternative strategies leverage quantum key distribution (QKD), which exploits quantum mechanics to secure communication channels inherently susceptible to quantum advancements [9].

QKD functions on the principle of securely distributing cryptographic keys between communicating parties [10,11], addressing the confidentiality aspect of security. However, current QKD protocols predominantly focus on confidentiality, prompting a strategic reassessment of cryptographic methodologies. This involves integrating QKD with PQC to address identity authentication and message integrity, thus forming a comprehensive cryptographic framework capable of withstanding evolving security challenges [12,13,14].

In classical cryptography, ensuring message confidentiality, identity authentication, and message integrity are primary objectives. Traditional cryptographic protocols, like QKD, primarily focus on ensuring message confidentiality through quantum principles for key distribution. However, current QKD protocols predominantly address only confidentiality, prompting a reevaluation of cryptographic methodologies. One innovative approach is to integrate QKD with PQC, which addresses identity authentication and message integrity challenges posed by both classical and quantum computers. By combining QKD and PQC, a comprehensive cryptographic framework emerges, fortifying classical cryptographic protocols against evolving security challenges and quantum threats.

To authenticate the classical channel within QKD, leveraging post-quantum algorithms is proposed to ensure the highest degree of quantum resistance. This study explores the interplay between PQC and QKD across various scenarios, introducing a novel deferred authentication approach. Investigating its efficacy and implications for security in quantum communication, the study draws parallels with classical cryptography, where security bits are generated during the key exchange process. By scrutinizing each algorithm’s implementation, the study enhances our understanding of the synergies between PQC and QKD, contributing to fortifying quantum communication against emerging threats.

QKD serves as a foundational pillar of security, deriving its strength from the principles of quantum mechanics [10,11]. Its reliability extends to practical systems, ensuring consistent security [15]. QKD has undergone significant evolution, particularly in real-world applications. Notably, secure key rates have surged to 26.2 Mbps, even in the face of a 4 dB channel loss, equivalent to the length of a 20 km optical fiber [16]. Moreover, practical optical fibers have demonstrated the capacity to distribute keys over distances exceeding 500 km [17,18]. Noteworthy achievements include QKD facilitated by the Micius satellite, spanning a distance of 1120 km [19]. These milestones are complemented by the establishment of diverse quantum networks, prominently the expansive network spanning 7600 km [20].

The pioneering research of Claude Shannon illustrated the possibility of achieving genuinely secure communication exclusively through one-time pad encryption, a method reliant on the establishment of a shared symmetric key between communicating entities. This necessitates a secure channel for distributing the key, ensuring its confidentiality and integrity. Upon possessing this shared key, both parties can engage in message encryption and decryption with utmost security. Throughout the key distribution process, verifying the authenticity of each party’s identity is paramount, typically accomplished through robust authentication protocols. However, traditional encryption and authentication techniques lack concrete security guarantees and are susceptible to exploitation by quantum algorithms, notably Shor’s algorithm [21], when applied in quantum computing environments. PQC [22] emerges as a promising avenue, offering cryptographic solutions resilient to attacks from Shor’s algorithm. Nonetheless, PQC’s resilience against other classical or quantum algorithms beyond Shor’s algorithm remains uncertain, highlighting the need for further exploration. While PQC may serve short-term security needs like authentication admirably, its long-term viability, particularly in information encoding, remains uncertain. To address this uncertainty, they propose an innovative approach that combines the strengths of PQC and QKD. By leveraging PQC for short-term authentication security and QKD for long-term key security, we establish a robust framework for secure communication. This facilitates the use of symmetric keys and one-time pad encryption, ensuring the satisfaction of both short-term and long-term security requirements [23] with the quantum bit error rate (QBER) of less than 0.5%.

Efforts to standardize PQC, which operates efficiently on current classical computers without necessitating specialized hardware, are presently underway within leading international standards bodies such as the NIST (National Institute of Standards and Technology). While considered a stopgap measure, there remains a minute probability that a novel algorithm could emerge, akin to Shor’s algorithm compromising RSA codes. Nonetheless, there is a growing recognition that QKD and PQC can synergize to offer a holistic solution. QKD techniques excel in highly secure, point-to-point communication links, while PQC exhibits versatility across a broad spectrum of security software applications. Despite the limited deployment of long-range QKD networks, there is an imperative to comprehensively discern their vulnerabilities to a myriad of attacks through standardized benchmarking and testing protocols before integrating them into critical infrastructure sectors. Concurrently, alongside technology and standards development, a concerted effort is required to pinpoint and cultivate real-world applications, drawing inspiration from pioneering initiatives like the European OPENQKD project, aimed at catalyzing widespread adoption of quantum cryptography.

In the past decade, QKD has experienced active and consistent progress in scientific research, application exploration, and industrial development, emerging as one of the most influential and practical quantum information technologies. Several national strategies in quantum science and technology have identified the QKD network as pivotal for realizing the future quantum Internet [24], harnessing its benefits for network information security assurance [25,26]. During this period, numerous innovative QKD protocols and implementations have been continuously refined, resulting in significant breakthroughs in system performance metrics such as maximum transmission distance and secure key rate [27,28]. Efforts have also been directed towards exploring the integrated deployment and flexible networking of QKD with Information and Communication Technology (ICT) systems and networks [29]. Furthermore, various types of QKD systems and encryption solutions have been commercialized by multiple vendors and service providers [30]. Globally, QKD network construction and demonstration application projects, primarily supported by public research and development funds, have been underway in numerous countries and regions [31]. This sustained innovation, application exploration, and commercialization endeavor underscores the significance of QKD-based Quantum Secure Communication (QSC) technology in the impending quantum era. Such advancements have garnered widespread recognition and appreciation from government, academia, and industry stakeholders.

In this paper, fundamental definitions of both quantum mechanics and cryptography based on the QKD-SARG04 and BB84 protocols are explained. It extends to encompass essential concepts intrinsic to classical cryptography, strategically positioned to complement the subsequent exploration of quantum counterparts. This comprehensive foundation lays the groundwork for a holistic understanding of the interdisciplinary intersection between quantum mechanics and cryptography. The methodology employed in this study takes center stage, where the intricate details of the research approach, experimental design, and analytical frameworks can be unveiled. The culmination of applied methodologies and investigative efforts effectively manifests itself in the crucible of empirical findings, showcasing the outcomes, trends, and observations derived from the meticulous implementation of the proposed methodology. The denouement of this work unfolds, where the conclusions drawn from the study are comprehensively discussed. Beyond the retrospective analysis, this work explores the broader implications of the findings and delves into prospective avenues for future research. It serves as a reflective platform, offering a synthesis of insights gleaned from the research endeavor and paving the way for ongoing discourse and exploration within the field. In sum, this organizational framework ensures a logical progression, guiding readers through the foundational concepts, methodological intricacies, and empirical revelations, and ultimately concluding with a thoughtful reflection on the broader implications and potential trajectories for future research.

## 2. Quantum Cryptography and Quantum Key Distribution

The objective of cryptography is to securely transmit confidential information across insecure communication channels, safeguarding against potential eavesdropping and unauthorized access. Encryption plays a pivotal role in ensuring data security, often achieved through the utilization of pre-shared secret keys. The feasibility of achieving absolute security through such methods has been demonstrated, particularly with the use of a one-time pad (OTP) [32].

In the OTP scenario, two users, Alice and Bob, possess a key consisting of perfectly correlated bits, ensuring confidentiality during message transmission. The key is applied through a bitwise XOR operation with the message, resulting in a randomized bit string that is transmitted through an insecure channel. This process ensures that the transmitted message remains indecipherable, provided the key is used only once. However, securely distributing the key poses a significant challenge, as demonstrated by Claude Shannon’s recognition of the key distribution problem [32]. While classical channels are inadequate for secure key exchange, leveraging quantum channels offers a solution. Quantum mechanics’ unique features enable the transmission of bits with guaranteed confidentiality, as affirmed by the no-cloning theorem [14]. This theorem, coupled with quantum states’ inherent collapse upon measurement, forms the foundation of quantum cryptography.

QKD protocols facilitate secure key distribution, encompassing various approaches like prepare-measurement protocols such as BB84 [10] and entanglement-based protocols like E91 [11]. This study focuses on simulating the SARG04 and BB84 protocols [33].

## 3. QKD Protocols

Utilizing the photon of light as a foundation, it is possible to develop protocols in discrete-variable or continuous-variable formats, each regarding light either as discrete or continuous photons. Discrete-variable QKD protocols capitalize on the particle aspect of light, encoding information within single photon states. Conversely, continuous-variable QKD protocols leverage the wave nature of light, encoding information within its amplitude and/or phase.

Discrete-variable QKD schemes come in two main types: Prepare and Measure (PaM) protocols including BB84 [10] and SARG04 [33] and Entanglement-Based (EB) protocols, including E91 [34] and BBM92 [35]. The earliest QKD protocols employed the PaM method, where a qubit state is generated and sent to the recipient party. It is worth noting that, in quantum computing, a quantum bit (qubit) represents a fundamental departure from classical bits and exists in a superposition of both 0 and 1 states simultaneously. Subsequently, EB protocols were introduced, allowing two parties to establish a secret key by performing measurements on a shared quantum state [36]. Unlike PaM protocols, EB protocols do not necessitate one communicating node to possess the joint state source or trust it. Instead, quantum correlations between measurements made by legitimate parties on the joint states can be examined using Bell’s theorem inequalities. While EB protocols offer enhanced security by eliminating the need for a trusted quantum source, PaM protocols remain more prevalent due to their simplicity. In this study, the SARG04 protocol in comparison with the BB84 one is effectively utilized.

### 3.1. BB84 Protocol

The theoretical unconditional security provided by QKD is capable of fulfilling communication security needs. The pioneering QKD protocol, proposed by Bennett and Brassard in 1984 (BB84) [10], has spurred significant theoretical and empirical research interest due to its foundational role in ensuring secure communication by leveraging the principles of quantum mechanics. The BB84 protocol’s ability to detect eavesdropping and ensure secure key exchange has made it a cornerstone of QKD research and development. Theoretical advancements have focused on reducing protocol complexity and enhancing system security. The B92 protocol [37] and the six-state protocol [38] are simplified and improved iterations of the BB84 protocol. The decoy-state protocol [39,40,41] further refines the BB84 protocol, enabling resistance to photon number-splitting (PNS) attacks. To date, real QKD systems have also been developed, achieving QKD in free-space air channels [42,43] and in optical fiber channels [44,45].

Our study introduces the concept of mono-authentication within QKD-BB84 protocol, a significant innovation aimed at streamlining the authentication process. Unlike traditional methods that authenticate at multiple stages, mono-authentication consolidates the authentication step to the end of the communication session. This not only simplifies the process but also minimizes potential vulnerabilities that adversaries could exploit during intermediate authentication steps. By focusing on a single authentication instance, mono-authentication enhances the overall security and efficiency of QKD systems.

### 3.2. SARG04 Protocol

The BB84 protocol uses the polarization of photons to create a shared secret key between two parties. The BB84 protocol [46] stands out as one of the most extensively employed QKD schemes renowned for its robust security mechanisms facilitated by the utilization of non-orthogonal states and random basis selections. However, its drawback lies in the protracted key exchange durations, rendering it less efficient. While BB84 exhibits moderate scalability, rendering it suitable for small-scale applications, its utility in larger networks might be limited.

SARG04, founded by Valerio Scarani, Antonio Acín, Gregoire Ribordy, and Nicolas Gisin, represents a 2004 quantum cryptography protocol being evolved from the original protocol of its sort, BB84. The SARG04 protocol is specifically engineered to withstand the photon number splitting attack [33]. Subsequently, an entangled version of the SARG04 protocol showcases its superiority over BB84 in terms of long-distance communication, resilience against Eve’s attack, and secret key rate [47]. The SARG04 protocol creates the n-state protocol, which maintains its reliance on two non-orthogonal quantum states [48].

A SARG04 protocol that uses the time-bin encoding of photons to create a shared secret key between two parties. The SARG04 protocol demonstrates robust resistance against PNS attacks. Similar to the B92 protocol, SARG04 employs two non-orthogonal quantum states. However, SARG04 encodes the bit in the basis rather than the state. Notably, in contrast to BB84, Alice refrains from disclosing her chosen basis to Bob. During the sifting phase, Bob discloses the bits he measured from the received qubits. If a bit revealed by Bob differs from the corresponding bit sent by Alice, it indicates that they utilized different polarization bases for preparation and measurement. In such instances, Alice instructs Bob to accept the bit, and Bob assigns the bit value associated with the unused basis during that measurement. This protocol was subsequently extended to n quantum states [49]. As a result, the SARG04 protocol boasts high security measures and enhanced efficiency compared to BB84, attributed to its utilization of only two states. Nonetheless, its scalability is relatively limited, thereby constraining its applicability within larger network infrastructures.

Both the SARG04 and BB84 protocols entail similar phases of transmission and measurement, with congruent initial steps. However, a pivotal distinction emerges in the subsequent phase. Here, Alice designates a pair of non-orthogonal states [50]. Rather than directly disclosing her chosen bases, she employs one of them to encode each bit. Bob then cross-references his bases for the corresponding bits. If Bob selects the appropriate base, he accurately measures the state; otherwise, he fails to retrieve the bit [33]. The SARG04 protocol has played a crucial role in establishing the security of photon pulses [51]. Moreover, in scenarios characterized by a weak signal generated by a Poissonian source and received by an imprecise detector, the SARG04 protocol proves to be particularly effective [52].

### 3.3. Quantum Bit Error Rate

For the Quantum Bit Error Rate (QBER) [53] in the context of this protocol, there exists a predefined threshold for acceptable errors, capped at 11% [54]. Should the QBER surpass this threshold, it signals a potential intrusion or tampering attempt by an eavesdropper during the communication process. Understanding and monitoring the QBER is crucial for maintaining the security of quantum communication. The calculated QBER serves as an indicator, helping ensure that the quantum key exchange remains robust against potential adversarial interventions.

Bob transmits the calculated QBER value to Alice. Independently, Alice performs her own QBER calculation. The success of the process is contingent upon both parties arriving at the same QBER value, confirming that the quantum communication is intact and secure. If the calculated QBER is below the predefined threshold of 11%, the protocol is deemed successful, and the sifted key is established.

In our quantum communication framework, we utilize the QBER as a critical metric to discern potential eavesdropping activities. Specifically, within the BB84 protocol, we adhere to a stringent constraint, allowing a maximum QBER of 11%. This threshold corresponds to the Holevo bound, indicating the maximum amount of classical information that can be reliably transmitted through a quantum channel. The introduction of a third party into the quantum channel, establishing an entangled state within its Hilbert space, poses a potential vulnerability. This scenario arises when Bob’s measurement indirectly prepares the eavesdropper’s state, thereby revealing certain information about the quantum states transmitted by Alice. Mathematically, the 11% threshold represents the point at which the allowed errors are capped. This is crucial to ensure that the mutual information between Alice and Bob remains greater than the information shared between Alice and the eavesdropper. Staying below this 11% threshold is imperative to maintain the security of the key exchange, as surpassing it would indicate a compromise in the integrity of the communication, prompting the need to discard the keys.

## 4. Strengthening QKD: Advancing Security through Post-Quantum Cryptography

In the post-processing phase of quantum communication, conducted within the conventional channel accessible to the public, users must prioritize verifying the authenticity of their communication partners and ensuring the integrity of public messages to prevent unauthorized alterations. To address this security vulnerability, we recommend incorporating authentication measures to establish a stronger and more secure framework, thereby protecting against potential threats to the integrity of quantum communication.

Authenticating the classical channel in QKD protocols entails various methods. However, some rely on pre-distributed keys from the initial round, which poses scalability challenges [55,56]. An optimal solution involves utilizing a Public Key Infrastructure (PKI). In this approach, trust is centralized in a Certificate Authority (CA), eliminating the need for individual user trust. The CA manages the distribution of public and private keys for each user, facilitating effective authentication through signature and verification algorithms. This PKI-based approach enhances scalability and trust within the QKD protocol.

Digital signatures are crucial for verifying the authenticity of transmitted data. This scheme comprises three key components: the key-pair, consisting of public and secret keys generated through a key generation algorithm; the signing algorithm, which creates the signature; and the verifying algorithm, which determines the success or failure of the verification process given the public key and message. These signatures guarantee the integrity and origin of exchanged data, thereby affirming the authenticity of the key exchange.

Signatures play a pivotal role in ensuring information–theoretic security within QKD. As long as the authentication process remains uncompromised during communication, QKD security is maintained at an information–theoretic level. This security remains resilient against decryption attempts, even if the public key signature is deciphered later [57]. Thus, reliance on the public key signature emerges as a singular and crucial element in the overall security framework. It is essential to emphasize that when referring to signatures, we encompass the entire verification process.

The effectiveness of digital signatures depends on the complexity of a mathematical challenge. This challenge, when combined with the right public and private keys, is easily solvable, but its difficulty escalates without the correct keys. In our efforts, we utilize exclusively post-quantum algorithms, which are based on mathematical problems resistant to decryption attempts by quantum computers. These algorithms are chosen for their ability to withstand cryptanalytic attacks enabled by quantum computing capabilities.

We now introduce mathematical challenges that pose significant hurdles even for quantum computers, along with the algorithms designated for inclusion in this study. All these algorithms are finalists in the National Institute of Standards and Technology (NIST) competition, strategically designed to standardize optimal algorithms in response to the looming threat posed by quantum computers. The selection process involves rigorous evaluation through multiple rounds of analysis. It is important to note that while these algorithms currently demonstrate resilience against decryption attempts, they face the challenge of potential vulnerabilities emerging from future cryptanalysis. Recently, one algorithm encountered a security breach, prompting a reevaluation of its robustness, highlighting the dynamic nature of cryptographic landscapes and the continuous effort to stay ahead of emerging threats.

Our initial focus is on lattice-based cryptosystems, renowned for their well-rounded performance. Notable algorithms grounded in this mathematical problem include CRYSTALS-DILITHIUM [58] and FALCON [59], which have officially been announced by NIST to standardize them. The prominence of lattice-based cryptosystems lies in their believed effectiveness across various cryptographic metrics, positioning them as robust contenders in the evolving landscape of post-quantum cryptographic solutions.

Another mathematical problem under consideration is multivariate-based cryptography, which relies on solving systems of multivariate polynomial equations. RAINBOW was initially a candidate rooted in this problem domain [60]. However, it is crucial to note, RAINBOW has been eliminated from consideration. In cryptographic terms, a primitive is considered “broken” when an attack compromises its security level, failing to uphold its advertised robustness. This elimination underscores the rigorous evaluation process and the commitment to ensuring cryptographic solutions maintain their advertised levels of security.

In conclusion, we shift our focus to hash-based cryptosystems, which offer one-time signature schemes based on hash functions and the security assumptions of one-way functions. An exemplary solution in this domain is SPHINCS+ [61], which has officially been announced by NIST. Hash-based cryptosystems, leveraging the robustness of one-time signature schemes, contribute to the evolving landscape of post-quantum cryptographic solutions, providing an alternative approach to address security challenges posed by quantum advancements. A critical consideration is ensuring the resilience of authentication algorithms to prevent unauthorized access for at least the number of bits exchanged. The concept of the security level, indicating the efficacy of signature algorithms, encapsulates this necessity. For an exchanged key of n bits, the security level mandates that an attacker would need to perform $2^{n}$ operations to successfully guess the key.

The post-quantum signature algorithms utilized in this study, CRYSTALS-DILITHIUM, SPHINCS+, and RAINBOW, are selected to offer distinct security levels. We explore security levels equivalent to 128, 192, and 256 bits of security. Notably, CRYSTALS-DILITHIUM and RAINBOW offer algorithms catering to all these security levels. Each algorithm’s name, contingent on the security level, is detailed in Table 1. This meticulous consideration of security levels underscores the commitment to fortifying the authentication process against potential breaches.

Having established the foundational concepts, we now turn our attention to the methodology and contributions of this work based on QKD authentication model integrated with PQC. Our forthcoming analysis focuses on a comparative evaluation of the performance exhibited by the four post-quantum algorithms introduced earlier. To provide additional insights, Table 2 presents variations in sizes among various parameters generated by these algorithms. This comparative assessment aims to uncover nuances in their performance characteristics, contributing to a deeper understanding of their applicability and effectiveness in quantum-resistant authentication.

Table 2 presents the sizes of signatures and key pairs for each post-quantum algorithm under examination. This table offers a comprehensive overview of key characteristics inherent to the studied algorithms. Notably, it highlights differences, particularly in the signature size for RAINBOW compared to other algorithms. Similarly, distinctions are observed in the key pair size for SPHINCS+. These nuances provide insights into the unique attributes and performance variations among the selected PQC algorithms.

## 5. QKD Authentication Model Setup and Details

In this study, our primary objective is to ascertain the most effective method for authenticating the classical channel within the BB84 and SARG04 protocols, leveraging the capabilities of post-quantum signature algorithms. Additionally, our focus extends to a detailed exploration of specific scenarios, leading us to formulate a set of recommendations tailored for practical implementation in a BB84 and SARG04 experimental QKD setups. To achieve this goal, we employ Python to simulate the procedural intricacies of the BB84 and SARG04 protocols.

A comprehensive breakdown of the technical aspects of this simulation is provided, offering an in-depth understanding of the simulation’s intricacies and methodologies. This simulation serves as a pivotal tool in our quest to refine classical channel authentication within the BB84 and SARG04 frameworks and extract practical insights for real-world QKD implementations. To achieve the requested outcomes, we conduct simulations of the QKD protocols, specifically the BB84 and SARG04 protocols. Our simulation will be implemented in a Python3 script. The Python3 script from Github.com encapsulates the entire simulation process, encompassing the BB84 and SARG04 protocols, classical channel authentication, error correction, and privacy amplification. Within this repository, one can also find the data processing procedures employed to generate the plots presented in this work. This open-access repository serves as a valuable resource for those interested in exploring the intricacies of the simulation and reproducing the results outlined in this study.

Initially, our exploration mirrors a process akin to classical cryptography, involving a key exchange. Within this framework, the QKD protocol is invoked to generate a predetermined number of bits, serving as the foundation for a subsequent symmetric key. The focus then shifts to evaluating the efficacy of various algorithms in authenticating the classical channel within the BB84 and SARG04 protocols. To scrutinize the performance of these algorithms, we meticulously examine specific variables across different security levels. This comprehensive analysis encompasses a thorough investigation of the variables under consideration throughout this study.

### 5.1. Mono-Authentication

**Figure 1:**
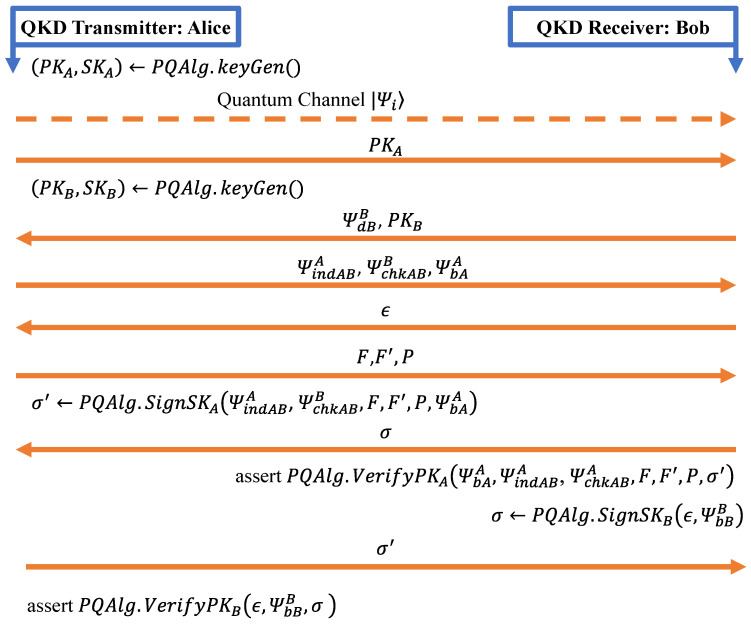
The signature style within the BB84 protocol is characterized by several key components [57]. In terms of communication, a visual representation is provided through the use of continuous orange arrows denoting information sent via the classical channel. Conversely, dotted orange arrows signify communication through the quantum channel, emphasizing the exchange of quantum information. The PQC algorithm is used to sign the Alice and Bob message digest and the nonce under their respective private keys to generate signatures.

Our initial exploration delves into determining the optimal steps within the protocols for authentication. In the sole authenticated QKD experimental implementation currently available [23], the implemented signature scheme involves signing and verifying at multiple stages of the protocol [57]. This investigation aims to discern the most effective points within the protocol for the authentication process. Mono-authentication, as defined in this context, refers to a process where authentication unfolds at various stages throughout the communication. In Figure 1, we pinpoint precisely where these signature operations take place. This approach involves incorporating authentication mechanisms at multiple steps to enhance the overall security and integrity of the communication process. We advocate for a mono-authentication style, wherein the signature generation occurs after the key exchange, concluding the communication. This approach involves Alice signing all the information she has transmitted to Bob, sending it to Bob for verification, and vice versa.

Bob communicates his readiness to Alice by transmitting the basis in which he has measured each bit, denoted as $\Psi_{bB}^{B}$. Subsequently, Alice initiates the crucial process of basis sifting. This involves Alice identifying and retaining bits in her bit string, $\Psi_{dAB}^{A}$, that align with Bob’s chosen basis, while discarding those with a mismatched basis. Following this initial sifting, Alice generates a new random bit string, $\Psi_{indAB}^{A}$. This string plays a pivotal role in determining which bits Alice will make public, forming $\Psi_{chkAB}^{A}$, and which bits she will preserve as a secret key, denoted as $\Psi_{kAB}^{A}$. Specifically, $\Psi_{chkAB}^{A}$ encompasses the substring of $\Psi_{dAB}^{A}$ for which the corresponding bits in $\Psi_{indAB}^{A}$ are set to 1, while $\Psi_{kAB}^{A}$ is composed of bits where the indicator is 0. Upon completing this intricate process, Alice publicly discloses $\Psi_{bAB}^{A}$, $\Psi_{indAB}^{A}$, and $\Psi_{chkAB}^{A}$. Importantly, Alice’s revelation of her basis choice at this stage holds significance, as Bob has already measured the transmitted qubits. This proactive measure ensures that quantum communication remains secure, guarding against potential interference from eavesdroppers.

Bob engages in a thorough comparison between Alice’s encoded bit string, $\Psi_{bAB}^{A}$, and his own measured counterpart, $\Psi_{bAB}^{B}$. During this process, he discards bits in his measured bit string, $\Psi_{dAB}^{B}$, where the basis choices deviate from Alice’s selections. Subsequently, leveraging the previously generated random bit string $\Psi_{indAB}^{A}$, Bob follows a procedure akin to Alice’s to obtain his sifted bit string components: $\Psi_{chkAB}^{B}$ (the revealed bit string) and $\Psi_{kAB}^{B}$ (the final secret key). It is noteworthy that the alignment of $\Psi_{chkAB}^{B}$ with $\Psi_{chkAB}^{A}$ is pivotal, as it allows Bob to determine the number of matching bits between the two. This comparison becomes the basis for estimating the QBER in the derived key. The QBER is defined as

(1) $$QBER=\frac{\left(\Psi_{chkAB}^{B0}-\Psi_{chkAB}^{A0}\right)+\left(\Psi_{chkAB}^{B1}-\Psi_{chkAB}^{A1}\right)}{n_{r}}\;$$

where

$\Psi_{chkAB}^{B0}$ is the number of 0 bits in $\Psi_{chkAB}^{B}$
$\Psi_{chkAB}^{B1}$ is the number of 1 bit in $\Psi_{chkAB}^{B}$
$\Psi_{chkAB}^{A0}$ is the number of 0 bit in $\Psi_{chkAB}^{A}$
$\Psi_{chkAB}^{A1}$ is the number of 1 bit in $\Psi_{chkAB}^{A}$

The length of the string containing the revealed bits is denoted as $n_{r}$, where $n_{r}=len\left(\Psi_{chkAB}^{i}|i=A\;or\;B\right)$ equals the length of the string.

At Alice’s error correction and privacy amplification stage, the primary objective is to securely exchange a secret key between Alice and Bob. The secret keys derived from previous steps are denoted as $\Psi_{kAB}^{A}$ and $\Psi_{kAB}^{B}$. Due to inherent noise in the communication channel, these keys may not match perfectly $\left(\Psi_{kAB}^{A}\neq \Psi_{kAB}^{B}\right)$. Our goal is to synchronize these keys to ensure they are identical. It is important to note that the original BB84 protocol did not account for noise in the communication channel, which led to the later introduction of error correction mechanisms. These mechanisms were added to rectify discrepancies and ensure the successful and secure exchange of the key between Alice and Bob.

Once it is confirmed that no eavesdropper has tampered with the quantum communication, the participants begin the error correction process. The goal is to align their respective strings, achieving the highest level of mutual information between Alice and Bob. Alice starts the error correction process by creating a hash of her secret key, $\Psi_{kAB}^{A}$, using the function F as:

(2) $$F:\;F’\;\leftarrow F(\Psi_{kAB}^{A})$$

F’ denotes the outcome of a secure hashing procedure, guaranteeing consistent results for specific inputs while preventing the original input from being inferred. This deterministic hashing is vital for synchronizing keys between Alice and Bob during error correction.

After error correction, the subsequent step is privacy amplification, a pivotal process aimed at bolstering key confidentiality. In mathematical terms, this entails minimizing the mutual information between Alice and Eve to the fullest extent possible. To accomplish this, Alice introduces a random permutation, denoted as P∈P, where P represents the set of permutations. Alice applies this permutation P to her secret key and then hashes the result using the same hash function utilized in the prior error correction phase. The resultant value, denoted as $\Psi_{skAB}^{A}=F\left(P(\Psi_{kAB}^{A})\right)$, signifies the ultimate secret key following privacy amplification. This method ensures the continued security of the key. The overall procedure unfolds as:

(3) $$Alice:\;\;\;\;P\in P\overset{F,\;P}{\to }Bob:\;\;\;\;\Psi_{skAB}^{A}∶=F\left(P(\Psi_{kAB’}^{B})\right)\;$$

At this point, Alice completes her part in the protocol. In the last phase, she communicates crucial details to Bob essential for error correction and privacy amplification. This involves sharing information such as the hash of the private key $\left(F’\right)$, the random permutation (P), and specifying the hash function she employed (F). Together, these elements equip Bob with the required parameters to execute the final protocol steps, guaranteeing the synchronization and security of the resultant secret key.

In the final phase of the key exchange, Bob initiates error correction and privacy amplification upon receiving the transmitted data from Alice. He begins by utilizing the hash $\left(F’\right)$ provided by Alice to conduct error correction. Bob computes the hash of his own key $\left(F^{\prime \prime }=F\left(\Psi_{kAB}^{B}\right)\right)$. A comparison between F’ and F″ is then made, and any disparity $\left(F’\neq F\prime \prime \right)$ indicates errors within the key. To rectify these errors, Bob systematically tests bit flips in his key until the computed hashes match. This meticulous process ensures alignment between the keys of Alice and Bob. Importantly, for potential eavesdroppers attempting to compromise the key, the task of brute-forcing a pre-image of the hash is impractical and computationally daunting. This robust approach underscores the security of the key exchange, instilling confidence in the unassailable integrity of the shared secret key.

To mitigate the computational burden associated with lengthy calculations, the implementation strategically limits the acceptable number of errors in the used key, a crucial parameter known as the maximum corrected errors. Bob systematically explores various combinations, initially attempting to flip one bit of his key at a time and comparing each potential option’s hash to F’. If this initial search fails, he repeats the process with two errors, continuing until conducting a brute force search capped at a predetermined maximum number of errors. If Bob exhausts all combinations without finding a hash match, he discards the key, triggering a restart of the QKD process—an abortive measure. Conversely, upon discovering matching hashes, he corrects these errors, resulting in the refined secret key denoted as $\Psi_{kAB’}^{B}.$ Subsequently, Bob proceeds with privacy amplification, culminating in the generation of the shared secret key $\Psi_{skAB}^{B}=F\;\left(P\left(\Psi_{kAB’}^{B}\right)\right)$, where $\Psi_{skAB}^{B}=\Psi_{kAB}^{A}$, indicating the successful exchange of a secret key utilizing quantum states.

A detailed schematic of this process is depicted in Figure 1, illustrating the sequential steps involved in this mono-authentication style. Within the mono-authentication approach, only two signatures are executed at the conclusion of the communication. An important consideration in this process is the error correction applied to the exchanged bit string. In scenarios where the key undergoes correction and the process is restarted, certain signatures may be discarded. In the context of mono-signature, emphasis is placed on the non-aborted instances, ensuring that only successfully completed signatures contribute to the authentication process.

First and foremost, the process begins with the invocation of PQAlg.KeyGen(), a function that generates both secret keys (SK) and public keys (PK) for users Alice and Bob. PQAlg specifically represents the chosen post-quantum algorithm implemented for authentication purposes. Integral to the authentication process are the signatures (σ), produced as outputs of the $PQAlg.{Sign}_{SK}()$ functions. These signatures play a crucial role and are transmitted through the classical channel. The subsequent verification step is pivotal. Utilizing $PQAlg.{Verify}_{PK}()$, the received signatures undergo scrutiny to determine their authenticity. The success of this authentication process dictates the seamless continuation of subsequent steps within the protocol. This comprehensive overview sheds light on the intricacies of the BB84 protocol’s signature style, encompassing key generation, communication modalities, signature generation, and the critical verification step that ensures the integrity of the authentication process.

Our authentication protocol incorporates a PK infrastructure enhanced with PQC algorithms, including CRYSTALS-DILITHIUM, SPHINCS+, and RAINBOW. Both the transmitter and receiver exchange certificates and sign the message with their private keys, subsequently verifying the signatures using public keys. To mitigate replay attacks, our mono-authentication protocol integrates the use of a nonce.

In summary, our approach leveraged cutting-edge PQC signature algorithms in tandem with a robust PK infrastructure to realize efficient and quantum-resistant authentication for QKD. The utilization of this advanced cryptographic technique not only ensures heightened security against potential quantum threats but also maintains the operational efficiency of QKD systems, such as key generation rates. By integrating PQC authentication, the conventional role of trusted relays within QKD networks can be supplanted by innovative optical switches, ushering in a new era of decentralized and streamlined connectivity. Each user’s interaction is streamlined through the acquisition of a singular digital certificate via the PK, enabling seamless direct links between any pair of users without the need for complex symmetric key distribution. Furthermore, the onboarding process for new network participants is simplified, requiring only the acquisition of a digital certificate rather than extensive key exchanges, thus facilitating swift integration into the QKD network fabric. In contrast to traditional pre-shared key methods, the adoption of PQC authentication brings tangible benefits in terms of operational simplicity and resource efficiency. Additionally, by reducing reliance on trusted relays, the security posture of the entire QKD network is fortified, ensuring resilience against potential vulnerabilities in the network infrastructure.

### 5.2. Quantum Communication Metrics: QBER, Maximum Corrected Errors, and Overhead

In our analysis, we introduce the concept of the probability ρ representing a random flip induced by the noise within the quantum channel. This probability aligns closely with the QBER, serving as an estimation of errors within the quantum channel. For the sake of simplicity, we treat QBER and ρ as interchangeable, assuming their equivalence for easier notation. The QBER holds significance in our study and serves as a crucial variable. To ground our investigation, we reference the experimentally estimated QBER in an authenticated QKD implementation [23]. Our examination of the QBER extends to an error threshold of 1.1%, the maximum value observed in experimental settings. Additionally, we delve into assessing the authentication cost concerning the time required for protocol execution, a metric we term as overhead. In certain instances, we break down the authentication process into its signature and verification components. This division allows for a more detailed comparison of the performance of each algorithm relative to the others. In such cases, we explicitly distinguish between mono-signatures/verifications, contributing to a nuanced analysis of algorithmic efficiency.

An additional parameter under consideration is the count of corrected errors. To manage computational resources effectively, we limit the correction process to address up to three errors. This decision is based on practical considerations, as a brute force search for four errors proves to be time-consuming. Details on the time required for worst-case error correction scenarios are presented in Table 3. Whenever the actual number of errors surpasses the predetermined limit for correction, the entire key associated with that exchange is discarded. Consequently, the exchange is aborted, and the protocol is restarted. The frequency of such aborts becomes another parameter of interest in our study, shedding light on the robustness and reliability of the key distribution process.

The failure time is computed for the error correction process. Table 3 presents the maximum (averaged) time that the script incurs in conducting a brute force search to address errors. This metric serves as an indicator of the computational overhead and time investment involved in rectifying errors during the error correction phase.

The count of hashes performed, presented in each cell, is derived from the combinatorial formula, which is used to determine the total number of unique hash operations conducted during the specified computational processes. This calculation offers insights into the computational complexity and hashing requirements associated with different scenarios and parameters.

(4) $$nC_{r}=\frac{n!}{r!\left(n-r\right)!}\;$$

The formula utilized for calculating the number of hashes performed is expressed as $nC_{r}$, where “n” represents the total number of bits under consideration (128, 192, or 256), and “r” corresponds to the maximum number of corrected bits. This formula provides a systematic approach to quantify the hashing operations required for various configurations, facilitating a detailed analysis of the computational demands involved.

In this context, it is important to note that the cumulative count of hashes includes those from the preceding rounds. For instance, in the scenario of a 128-bit key with a brute force search allowing up to one error, the process involves hashing the original string (1 hash) and subsequently hashing the 128 possible combinations corresponding to flipping only one bit (128 hashes). In total, this results in 129 hashes. This principle applies consistently across the different cases under consideration.

Having thoroughly examined the introduced parameters, our focus shifted to investigating how the quantity of exchanged keys influences the performance of the algorithms. This exploration entailed studying a practical QKD setup characterized by a continuous photon stream. Our approach involved periodic authentication of the exchanged bits within a predefined timeframe, guided by a specified key rate, rather than a static request for a specific number of bits at the initiation of each round.

### 5.3. Compact, Moderate, and Sizable Compartments

In our exploration of a continuous photon stream within the QKD protocol, we investigated how the authentication time for each post-quantum algorithm is affected by the size of the cryptographic key. Initially, we examined the temporal aspects associated with the authentication process of each algorithm across a range of key sizes, from 0 to 2000 bits. Subsequently, we categorized these outcomes into three distinct bins based on key size ranges. The small bin comprised key sizes between 0 and 100 bits, the medium bin covered the range from 100 to 500 bits, and the large bin encompassed key sizes from 500 to 2000 bits. This systematic categorization facilitated a detailed analysis, allowing us to compare algorithmic performance across scenarios characterized by varying key length requirements, from smaller to medium and larger keys.

### 5.4. Different Key Rates

In the final phase of our study, we explore how the designated key rate influences authentication costs during the continuous basis sifting process. Generating the sifted key poses the challenge of timing and frequency of authentication, with unique features for each post-quantum algorithm. Our strategy involves identifying the optimal period for each algorithm to minimize signature execution time within this continuous timeframe. To offer practical insights, we provide a detailed table outlining the recommended minimum signature times for each algorithm. This thorough guide empowers users to find the minimum waiting time between signatures, tailored to the specific demands of a given key rate. This approach enhances the understanding of the relationship between key rate dynamics and authentication efficiency, aiding informed decision-making in QKD setups.

## 6. Outcomes of QKD Mono-Authentication

**Figure 2:**
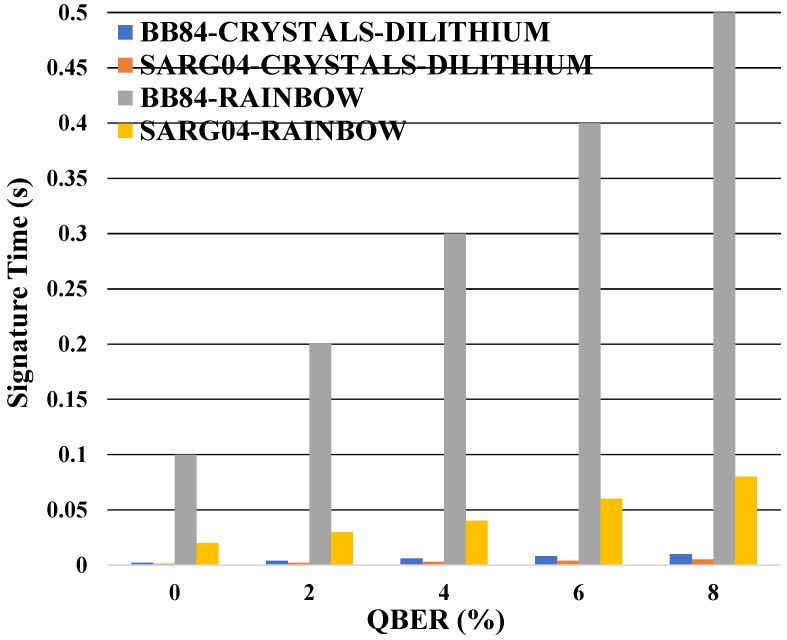
The signature time of mono-authentication as a function of the QBER for the two distinct protocols.

Findings are presented by concerning two transmission scenarios: block-based and continuous photon wave, which can be generated through a tunable laser, spontaneous parametric down conversion (SPDC), an optical parametric generator (OPG), or an optical parametric oscillator (OPO). Our analysis comprises 100 iterations for the mono-authentication algorithm based on the BB84 and SARG04 protocols. We evaluate the efficacy of various post-quantum algorithms in the mono-authentication method across different parameters. Initially, we analyze how the outcomes are influenced by the level of noise present in the quantum channel and then in continuous photon stream. Regarding the mono-authentication method, we observe in the plot that the SARG04 signature times are always slower than the BB84 signature ones as shown in Figure 2. For example, in the RAINBOW, we can observe that the SARG04 signature time is 0.08 s, while it is 0.5 s for the BB84 signature time.

**Figure 3:**
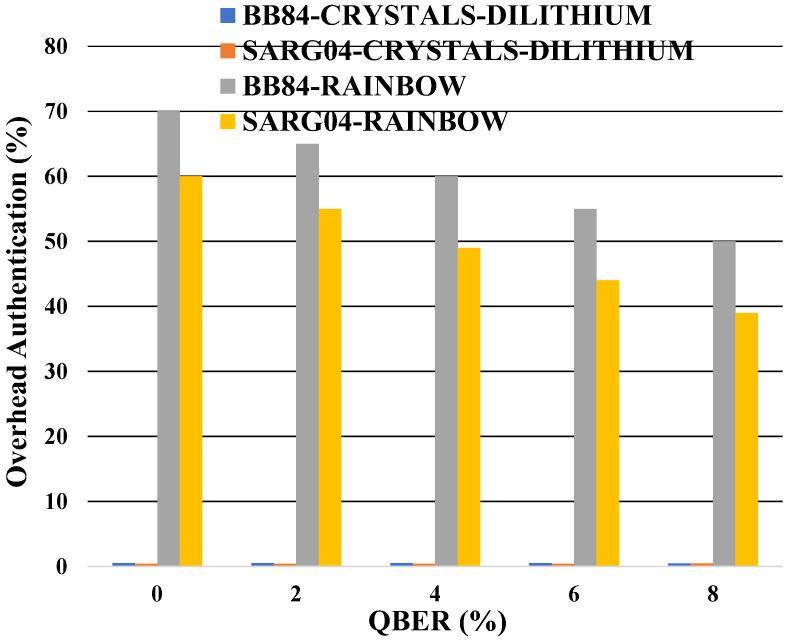
The overhead of the mono-authentication as a function of the QBER for two different protocols.

An intriguing finding is the consistent overhead of authentication in the mono-authentication configuration with CRISTALS-DILITHIUM, as depicted in Figure 3. For the BB84 protocol, this overhead remains at approximately 0.5%, while for the SARG04 protocol, it stands at 0.4%. However, there is a notable decrease in overhead for RAINBOW mono-authentication, dropping from 70 to 50% for the BB84 protocol and from 60 to 39% for the SARG04 one. It is worth noting that the security bits are 128 in this simulation. The following observations are made with the QBER fixed at 11%, representing the worst-case scenario observed experimentally.

**Figure 4:**
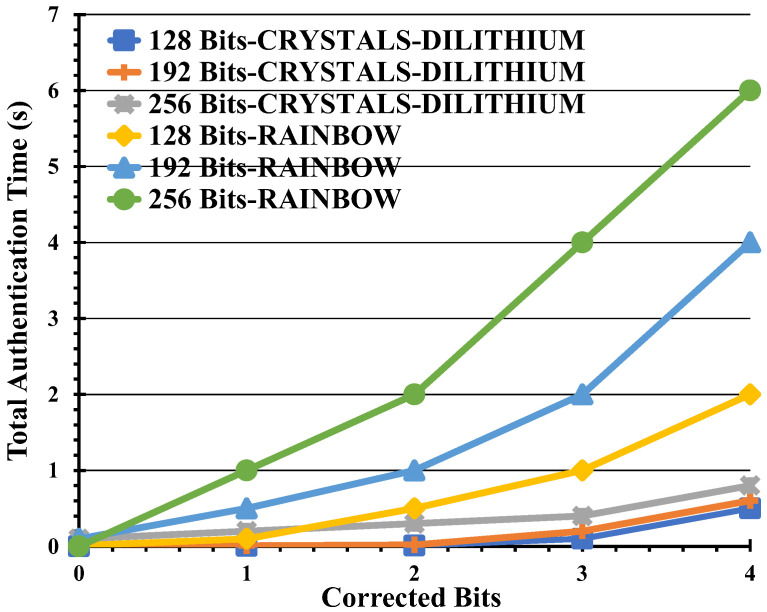
The graph illustrates the total authentication time, encompassing both signature and verification durations, alongside the time required for error correction, across varying maximum corrected bits given in Table 3. These data points are presented for three distinct security levels, all under a constant QBER of 11%.

We investigated the optimal threshold for maximum corrected errors within the mono-authentication algorithm. As depicted in Figure 4, we observed a notable increase in the total authentication time for the SARG04 protocol as the error correction bits escalated as well as with the security bits. Additionally, we computed the duration required to rectify a specific number of errors relative to the key size and noted a corresponding increase with the number of maximum corrected bits. The maximum durations for correcting between 0 and 3 errors across each security level are documented in Table 3.

**Figure 5:**
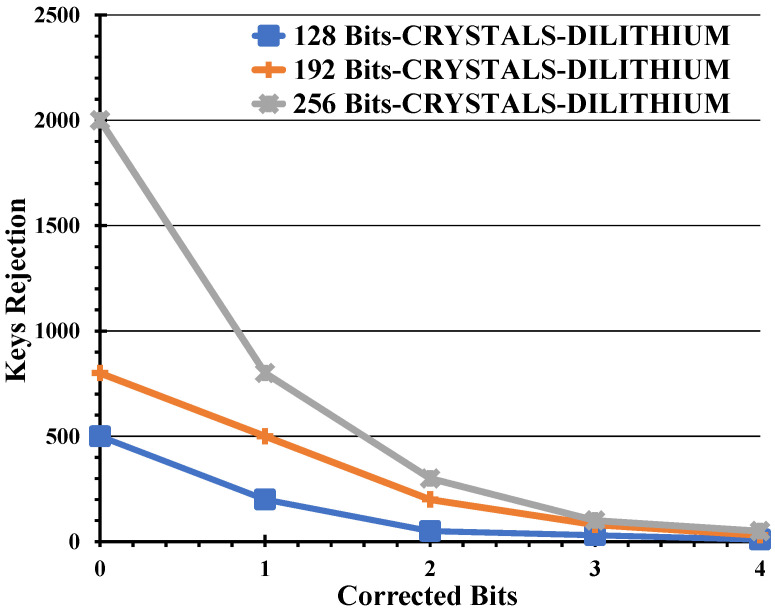
The varying security bits led to the key’s rejection, correlating with the number of bits selected for correction.

Examining Figure 5 sheds light on why authentication becomes more financially burdensome in cases where no corrections are made. The trend becomes evident: as the security level escalates, the number of rejected keys surges in the absence of error rectification. This surge in rejections is primarily attributed to the introduction of a QBER of 0.011. Moreover, longer keys inherently harbor a heightened likelihood of containing errors, amplifying the rejection frequency.

In this section, our inquiry delves into elucidating the minimum intervals essential for recommencing QKD authentication, contingent upon the bit rate of photons transmitted through the quantum channel. Nonetheless, prior to embarking on this investigation, it is imperative to ascertain the duration required by each algorithm to authenticate a designated quantity of bits.

**Figure 6:**
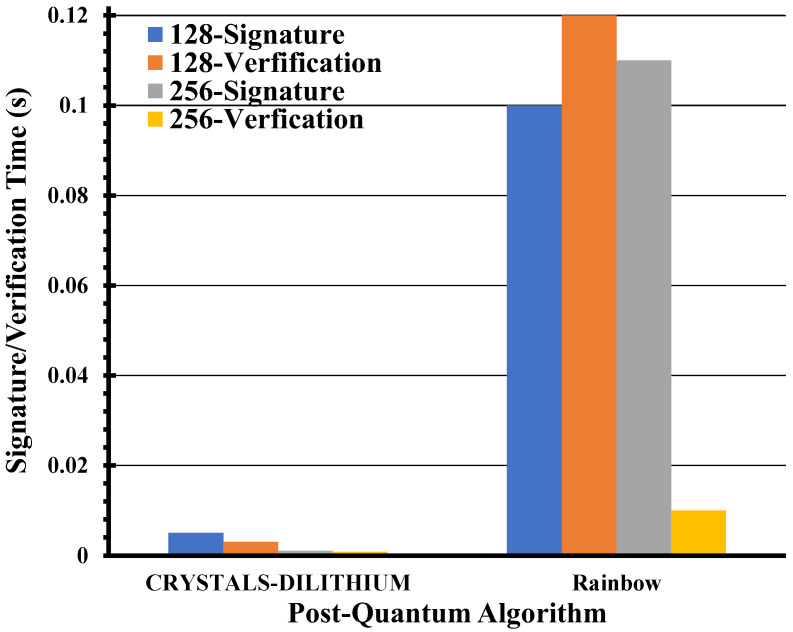
The plot illustrates the signature and verification times for two post-quantum algorithms, computed for the mono-authentication scheme based on the QKD-SARG04 protocol.

Figure 6 illustrates that CRYSTALS-DILITHIUM for mono-authentication for two different security bits 128 and 256 for the SARG04 protocol at the key rate of 50 kb/s exhibits the highest authentication rate, while RAINBOW performs the least efficiently. Interestingly, in most scenarios, the authentication performance appears to be largely unaffected by the number of bits being signed, particularly when signing up to 2000 bits. It is noteworthy that these results were derived after conducting a comprehensive analysis of the time taken by each algorithm to authenticate a range of bits, approximately from 0 to 2000, prior to averaging them to obtain the presented outcomes.

In Table 4, we determine the rate at which each algorithm can generate signatures per second relative to a given key rate. Since none of the algorithms complete an exchange of more than 2000 bits within the specified time intervals, continuous authentication remains feasible within those periods. Notably, CRYSTALS-DILITHIUM displays the highest throughput, while RAINBOW exhibits the slowest performance. For example, considering a key rate of 100 kbps, the minimal duration required to authenticate the transmitted data within the designated timeframe is 0.0011 s.

The mono-authentication approach significantly enhances authentication efficiency by delivering results swiftly. Noise serves as a critical factor amplifying the divergence in performance between verification and signature methods, as well as in overhead authentication. It is noteworthy that in noisy channel environments, mono-authentication emerges as the optimal implementation, as it remains unaffected by any QKD parameter. In contexts where the quantum channel incurs substantial costs, meticulous error scrutiny takes precedence. Conversely, in scenarios with exceptionally economical quantum channels, the preferred strategy often involves discarding keys and initiating fresh exchanges.

In our evaluation of post-quantum algorithms, CRYSTALS-DILITHIUM has consistently demonstrated outstanding performance in total authentication time, encompassing both signature and verification phases. Remarkably, CRYSTALS-DILITHIUM exhibits minimal overhead, consistently below 0.5% for both BB84 and SARG04 protocols. Further exploration across various security levels reveals a discernible trend: as authentication costs rise, processing durations extend. Consequently, transactions with lower security bit levels tend to feature shorter authentication times. To conclude, the analysis of recommendations across different key rates consistently highlights CRYSTALS-DILITHIUM’s superior performance over the RAINBOW post-quantum scheme.

**Figure 7:**
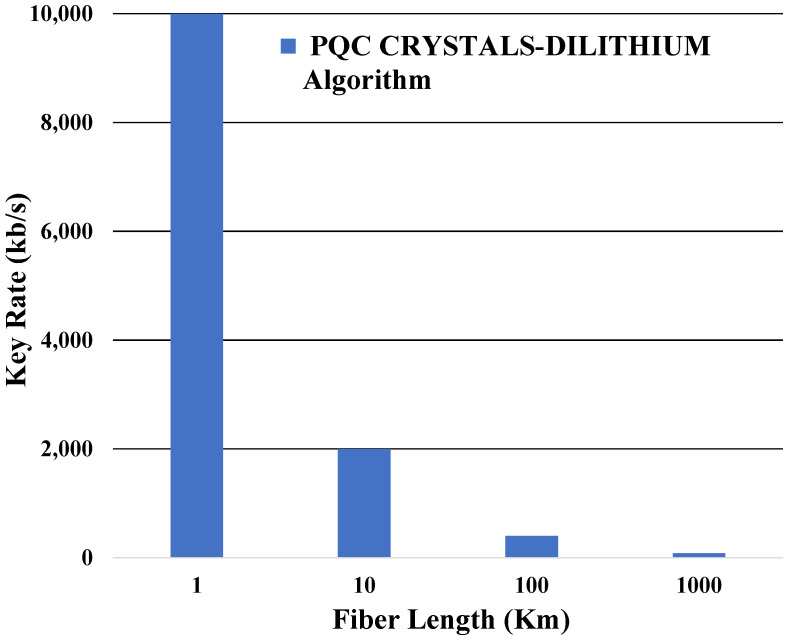
When QKD is authenticated with the PQC CRYSTALS-DILITHIUM algorithm, the secure key rate varies with the fiber length.

In the final phase, we successfully applied the PQC CRYSTALS-DILITHIUM algorithm within the context of a QKD point-to-point link, spanning fiber distances ranging from 1 to 1000 km. As depicted in Figure 7, the key rates exhibit a characteristic decline with increasing fiber length, aligning closely with theoretical predictions. To gain further insights, we conducted a comparative analysis of key rates across varying fiber lengths, employing both the QKD-BB84 and QKD-SARG04 protocols. Notably, the disparity in average key rates between the two protocols was found to be less than 0.5 standard deviations, indicating a high degree of consistency in their performance.

In contrast, we conducted a comparison between our theoretical QKD system, utilizing the PQC mono-authentication CRYSTALS-DILITHIUM algorithm, and an experimental PQC authentication method employing the Shor algorithm [23]. Our algorithm demonstrates a superior key rate of up to 10,000 kb/s and supports longer fiber lengths of up to 1000 km. However, it requires experimental validation, which constitutes a primary objective of our future research endeavors.

The impact of the proposed mono-authentication paradigm on QKD security encompasses its repercussions and influences on the security framework of QKD systems. Unlike conventional methods, mono-authentication streamlines the authentication process, consolidating it into a single step at the conclusion of communication. This consolidation offers potential benefits by simplifying authentication and minimizing vulnerabilities that adversaries could exploit. Evaluating the impact involves examining how the approach affects the overall security posture of QKD systems, considering its strengths, weaknesses, and implications for securing quantum communication.

While our study predominantly focuses on numerically analyzing post-quantum algorithms within mono-authentication scenarios in QKD protocols, the broader implications of our approach on QKD security warrant thorough exploration. Mono-authentication, as advocated in our study, represents a significant shift in the authentication process within QKD systems, optimizing efficiency by condensing authentication into a singular, conclusive step. This streamlined process not only enhances protocol efficiency but also introduces potential security benefits by reducing opportunities for adversary intervention during authentication. Furthermore, our findings highlight the superior performance of CRYSTALS-DILITHIUM and its efficiency in communication steps, bolstering the security and resilience of QKD implementations. Additionally, our study underscores the importance of considering real-world factors, such as noise in QKD scenarios, which may influence the effectiveness of authentication methods. Moving forward, exploring the broader implications of our approach on QKD security entails assessing its resilience against quantum attacks, scalability in large-scale QKD deployments, and compatibility with emerging quantum technologies. Through addressing these aspects, our aim is to not only advance QKD security but also contribute to the broader field of quantum cryptography, facilitating secure communication in the quantum computing era.

## 7. Conclusions

Our study has undertaken a comprehensive numerical analysis of two post-quantum algorithms, CRYSTALS-DILITHIUM and RAINBOW, selected from the NIST standardization process. We specifically focused on their performance within the mono-authentication scenario across varying security levels, examining block sizes of 128, 192, or 256 bits in both block-based and continuous photon transmission scenarios. Our investigation revealed the consistent superiority of mono-authentication, particularly within the QKD-BB84 and SARG04 protocols. Additionally, CRYSTALS-DILITHIUM demonstrated faster performance and greater efficiency compared to RAINBOW algorithms with excellent overheads and QBER for both protocols, highlighting its potential for enhancing QKD security. Our findings indicate that mono-authentication significantly reduces the cost and complexity of QKD, particularly in noisy environments, thereby paving the way for more robust and efficient quantum communication systems.

Furthermore, our analysis underscored the critical role of noise in realistic QKD scenarios, emphasizing the need to optimize error correction strategies as quantum channels improve. While our findings suggest promising avenues for improving QKD security, further exploration is warranted, particularly in assessing the scalability and applicability of mono-authentication in diverse computing environments. Looking ahead, there are several directions for advancing this research. One avenue involves establishing a security proof for the mono-authentication method to ensure its mathematical robustness. Alternatively, exploring the integration of hash functions presents an intriguing opportunity to enhance both security and efficiency in future studies. By addressing these avenues, we aim to contribute to the ongoing evolution of quantum cryptography and secure communication protocols [54,62].

A novel theoretical authentication paradigm termed mono-authentication is introduced, which incorporates CRYSTALS-DILITHIUM and RAINBOW algorithms within the QKD-SARG04 and BB84 protocols. This paradigm represents a significant departure from conventional methods, advocating for authentication solely at the conclusion of communication, thereby offering a streamlined solution. Additionally, we recognize the importance of comparative analysis in evaluating the efficacy of authentication methods in enhancing QKD security. Therefore, in future work, we plan to conduct an experimental comprehensive comparison between our mono-authentication paradigm and the proposed previous method [23], which may involve the Shor algorithm. Such analysis will provide insights into different authentication approaches’ strengths and weaknesses and their broader impact on QKD security. Through this comparative study, we aim to advance research in quantum cryptography and bolster the security of QKD systems.

## Figures and Tables

**Table 1:** Compilation of post-quantum algorithms and corresponding security levels.

Security Bits	DILITHIUM	SPHINCS+	FALCON	RAINBOW
128	DILITHIUM 2	SPHINCS-128f-simple	FALCON 512	RAINBOW IIIc-classic
192	DILITHIUM 3	SPHINCS-192f-simple	-	RAINBOW Vc-classic
256	DILITHIUM 5	SPHINCS-256f-simple	FALCON 1024	-

**Table 2:** The sizes in bytes for secret keys (SK), public keys (PK), and signatures (sig) across three distinct security levels, 128, 192, and 256 bits, are presented. These measurements apply to each post-quantum algorithm examined in this study. All the reported values ensure a reliable and standardized benchmark for performance assessment in the realm of post-quantum cryptography.

Post-Quantum Algorithm					
Security Bits	Size in Bytes	DILITHIUM	SPHINCS+	FALCON	RAINBOW
128	SK	2544	64	1281	626,048
	PK	1312	32	897	882,080
	Sig	2420	16,972	659	164
192	SK	4016	96	-	1,408,736
	PK	1952	48	-	1,930,600
	Sig	3293	35,664	-	212
256	SK	4880	128	2305	-
	PK	2592	64	1793	-
	Sig	4595	49,216	1276	-

**Table 3:** The maximum time in seconds, denoted in parentheses alongside the number of hashes performed, across three distinct security levels—128, 192, and 256—for varying maximum numbers of corrected bits. This analysis provides insights into the computational efficiency and resource requirements associated with different levels of error correction within the specified security contexts.

Maximum Corrected Bits				
Secuirty Bits	0	1	2	3
128	≈0.005 s (1 h)	≈0.022 s (129 h)	≈0.95 s (8257 h)	≈40 s (691,000 h)
192	≈0.006 s (1 h)	≈0.036 s (193 h)	≈3.5 s (18,529 h)	≈188 s (2,341,089 h)
256	≈0.007 s (1 h)	≈8 s (32,897 h)	≈8 s (32,897 h)	≈581 s (5,559,937 h)

**Table 4:** The minimum duration required by each post-quantum signature algorithm for mono-authentication, expressed in terms of key rate.

Post-Quantum Algorithms			
Secuirty Bits	Key Rate (kb/s)	CRYSTALS-DILITHIUM Time (s)	RAINBOW Time (s)
128	100	0.0011	0.11
192	1000	0.0024	0.14
256	10,000	0.0088	0.2

## Data Availability

Data underlying the results presented in this paper are not publicly available at this time but may be obtained from the authors upon reasonable request.

## References

[1] Grover L.K. (1996). A fast quantum mechanical algorithm for database search. arXiv.

[2] Shor P.W. (1997). Polynomial-Time Algorithms for Prime Factorization and Discrete Logarithms on a Quantum Computer. SIAM J. Comput..

[3] Proos J., Zalka C. (2004). Shor’s discrete logarithm quantum algorithm for elliptic curves. arXiv.

[4] Lloyd S. (1996). Universal Quantum Simulators. Sci. New Ser..

[5] Preskill J. (2012). Quantum computing and the entanglement frontier. arXiv.

[6] Arute F., Arya K., Babbush R., Bacon D., Bardin J.C., Barends R., Biswas R., Boixo S., Brandao F.G.S.L., Buell D.A. (2019). Quantum Supremacy using a Programmable Superconducting Processor. Nature.

[7] Rivest R.L., Shamir A., Adleman L. (1978). A Method for Obtaining Digital Signatures and Public-Key Cryptosystems.

[8] Miller V.S., Williams H.C. (1986). Use of elliptic curves in cryptography. Advances in Cryptology-CRYPTO ’85 Proceedings.

[9] Koblitz N. (1987). Elliptic curve cryptosystems. Math. Comput..

[10] Bennett C.H., Brassard G. (1984). Quantum Cryptography: Public Key Distribution and Coin Tossing.

[11] Ekert A.K. (1991). Quantum cryptography based on bell’s theorem. Phys. Rev. Lett..

[12] Bernstein D.J. (2009). Introduction to post-quantum cryptography. Post-Quantum Cryptography.

[13] Bernstein D.J., Lange T. (2017). Post-quantum cryptography. Nature.

[14] Wootters W.K., Zurek W.H. (1982). A single quantum cannot be cloned. Nature.

[15] Xu F., Ma X., Zhang Q., Lo H.-K., Pan J.-W. (2020). Secure quantum key distribution with realistic devices. Rev. Mod. Phys..

[16] Islam N.T., Lim C.C.W., Cahall C., Kim J., Gauthier D.J. (2017). Provably secure and high-rate quantum key distribution with time-bin qudits. Sci. Adv..

[17] Chen J.-P., Zhang C., Liu Y., Jiang C., Zhang W., Hu X.-L., Guan J.-Y., Yu Z.-W., Xu H., Lin J. (2020). Sending-or-not-sending with independent lasers: Secure twin-field quantum key distribution over 509 km. Phys. Rev. Lett..

[18] Fang X.-T., Zeng P., Liu H., Zou M., Wu W., Tang Y.-L., Sheng Y.-J., Xiang Y., Zhang W., Li H. (2020). Implementation of quantum key distribution surpassing the linear rate-transmittance bound. Nat. Photonics.

[19] Yin J., Li Y.-H., Liao S.-K., Yang M., Cao Y., Zhang L., Ren J.-G., Cai W.-Q., Liu W.-Y., Li S.-L. (2020). Entanglement-based secure quantum cryptography over 1120 kilometres. Nature.

[20] Liao S.-K., Ca W.-Q., Handsteiner J., Liu B., Yin J., Zhang L., Rauch D., Fink M., Ren J.-G., Liu W.-Y. (2018). Satellite-relayed intercontinental quantum network. Phys. Rev. Lett..

[21] Shor P.W., Goldwasser S. Algorithms for quantum computation: Discrete logarithms and factoring. Proceedings of the 35th Annual Symposium on Foundations of Computer Science.

[22] Chen L., Jordan S., Liu Y.-K., Moody D., Peralta R., Perlner R., Tone D.-S. (2016). Report on Post-Quantum Cryptography.

[23] Wang L.-J., Zhang K.-Y., Wang J.-Y., Cheng J., Yang Y.-H., Tang S.-B., Yan D., Tang Y.-L., Liu Z., Yu Y. (2021). Experimental authentication of quantum key distribution with post-quantum cryptography. NPJ Quantum Inf..

[24] Wehner S., Elkouss D., Hanson R. (2018). Quantum internet: A vision for the road ahead. Science.

[25] Kleese van Dam K. (2020). From Long-Distance Entanglement to Building a Nationwide Quantum Internet: Report of the DOE Quantum Internet Blueprint Workshop.

[26] Lewis A.M., Travagnin M. (2022). A Secure Quantum Communications Infrastructure for Europe: Technical Background for a Policy Vision.

[27] Wang S., Yin Z.-Q., He D.-Y., Chen W., Wang R.-Q., Ye P., Zhou Y., Fan-Yuan G.-J., Wang F.-X., Zhu Y.-G. (2022). Twin-field quantum key distribution over 830-km fibre. Nat. Photonics.

[28] Yuan Z.L., Plews A., Takahashi R., Doi K., Tam W., Sharpe A.W., Dixon A.R., Lavelle E., Dynes J.F., Murakami A. (2018). 10-Mb/s Quantum Key Distribution. J. Light. Technol..

[29] Aguado A., Lopez V., Lopez D., Peev M., Poppe A., Pastor A., Folgueira J., Martin V. (2019). The Engineering of Software-Defined Quantum Key Distribution Networks. IEEE Commun. Mag..

[30] FG QIT4N (Focus Group on Quantum Information Technology for Networks) (2021). Standardization Outlook and Technology Maturity: Quantum Key Distribution Network.

[31] Travagnin M., Lewis A.M. (2019). Quantum Key Distribution In-Field Implementations.

[32] Shannon C.E. (1949). Communication theory of secrecy systems. Bell Syst. Tech. J..

[33] Scarani V., Acin A., Ribordy G., Gisin N. (2004). Quantum cryptography protocols robust against photon number splitting attacks for weak laser pulse implementations. Phys. Rev. Lett..

[34] Ekert A.K. (1992). Quantum cryptography and Bell’s theorem. Quantum Measurements in Optics.

[35] Bennett C.H., Brassard G., Mermin N.D. (1992). Quantum cryptography without Bell’s theorem. Phys. Rev. Lett..

[36] Ecker S., Pseiner J., Piris J., Bohmann M. Advances in entanglement-based qkd for space applications. Proceedings of the SPIE, International Conference on Space Optics-ICSO.

[37] Bennett C.H. (1992). Quantum cryptography using any two nonorthogonal states. Phys. Rev. Lett..

[38] Bruß D. (1998). Optimal eavesdropping in quantum cryptography with six states. Phys. Rev. Lett..

[39] Lo H.-K., Ma X., Chen K. (2005). Decoy state quantum key distribution. Phys. Rev. Lett..

[40] Wang X.-B. (2005). Beating the photon-number-splitting attack in practical quantum cryptography. Phys. Rev. Lett..

[41] Ma X., Qi B., Zhao Y., Lo H.-K. (2005). Practical decoy state for quantum key distribution. Phys. Rev. A.

[42] Liao S.-K., Cai W.-Q., Liu W.-Y., Zhang L., Li Y., Ren J.-G., Yin J., Shen Q., Cao Y., Li Z.-P. (2017). Satellite-to-ground quantum key distribution. Nature.

[43] Yin J., Cao Y., Li Y.-H., Liao S.-K., Zhang L., Ren J.-G., Cai W.-Q., Liu W.-Y., Li B., Dai H. (2017). Satellite-based entanglement distribution over 1200 kilometers. Science.

[44] Yin H.-L., Chen T.-Y., Yu Z.-W., Liu H., You L.-X., Zhou Y.-H., Chen S.-J., Mao Y., Huang M.-Q., Zhang W.-J. (2016). Measurement device- independent quantum key distribution over a 404 km optical fiber. Phys. Rev. Lett..

[45] Boaron A., Boso G., Rusca D., Vulliez C., Autebert C., Caloz M., Perrenoud M., Gras G., Bussières F., Li M.-J. (2018). Secure quantum key distribution over 421 km of optical fiber. Phys. Rev. Lett..

[46] Wiesner S. (1983). Conjugate coding. ACM Sigact News.

[47] Yang L., Zhu Q., Li S., Ansari I.S., Yu S. (2021). On the Performance of Mixed FSO UWOC Dual-Hop Transmission Systems. IEEE Wirel. Commun. Lett..

[48] Branciard C., Gisin N., Kraus B., Scarani V. (2005). Security of two quantum cryptography protocols using the same four qubit states. Phys. Rev. A Gen. Phys..

[49] Koashi M. (2005). Security of quantum key distribution with discrete rotational symmetry. arXiv.

[50] Padamvathi V., Vardhan B.V., Krishna A.V.N. Quantum cryptography and quantum key distribution protocols: A survey. Proceedings of the 2016 IEEE 6th International Conference on Advanced Computing (IACC).

[51] Javed M., Aziz K. A survey of quantum key distribution protocols. Proceedings of the 7th International Conference on Frontiers of Information Technology.

[52] Fung C.-H.F., Tamaki K., Lo H.-K. (2005). On the performance of two protocols: SARG04 and BB84. arXiv.

[53] Zhao S., Li W., Shen Y., Yu Y., Han X., Zeng H., Cai M., Qian T., Wang S., Wang Z. (2019). Experimental investigation of quantum key distribution over a water channel. Appl. Opt..

[54] Ekert A., Christandl M., Renner R. (2004). A Generic Security Proof for Quantum Key Distribution.

[55] Fung C.-H.F., Ma X., Chau H.F. (2010). Practical issues in quantum-key-distribution post-processing. Phys. Rev. A.

[56] Kiktenko E.O., Malyshev A.O., Gavreev M.A., Bozhedarov A.A., Pozhar N.O., Anufriev M.N., Fedorov A.K. (2020). Lightweight authentication for quantum key distribution. IEEE Trans. Inf. Theory.

[57] Mosca M., Stebila D., Ustaoglu B. (2013). Quantum Key Distribution in the Classical Authenticated Key Exchange Framework. Proceedings of the Post-Quantum Cryptography: 5th International Workshop, PQCrypto 2013.

[58] Ducas L., Kiltz E., Lepoint T., Lyubashevsky V., Schwabe P., Seiler G., Stehlé D. (2018). Crystals-DILITHIUM: A lattice-based digital signature scheme. IACR Trans. Cryptogr. Hardw. Embed. Syst..

[59] Ducs L., Lyubashevsky V., Prest T. (2014). Efficient Identity-Based Encryption over Ntru Lattices.

[60] Ding J., Schmidt D. (2005). RAINBOW, a New Multivariable Polynomial Signature Scheme.

[61] Aumasson J.-P., Bernstein D.J., Beullens W., Dobraunig C., Eichlseder M., Fluhrer S., Gazdag S.-L., Hülsing A., Kampanakis P., Kölbl S. (2015). SPHINCS: Practical Stateless Hash-Based Signatures.

[62] Schwabe P., Stebila D., Wiggers T., Bertino E., Shulman H. (2021). More efficient post-quantum KEMTLS with pre-distributed public keys. Proceedings of the 26th European Symposium on Research in Computer Security (ESORICS).

